# Two Cases of Kounis Syndrome Caused by Contrast Medium: Early Extracorporeal Membrane Oxygenation

**DOI:** 10.1002/ccr3.72635

**Published:** 2026-04-28

**Authors:** Kiichi Yano, Yu Asami, Takumi Inoue, Emiko Takego, Kenji Tamai, Hiroyuki Takahashi

**Affiliations:** ^1^ Department of Anesthesiology Toho University Omori Medical Center Tokyo Japan; ^2^ Department of Intensive Care Shonan‐Fujisawa Tokushukai Hospital Fujisawa Japan; ^3^ Department of Intensive Care Saiseikai Yokohamashi Tobu Hospital Yokohama Japan

**Keywords:** anaphylaxis, cardiac arrest, coronary artery bypass, extracorporeal membrane oxygenation, Kounis syndrome, ventricular fibrillation

## Abstract

In cardiology, Kounis syndrome (KS), also known as allergic angina or myocardial infarction, is often overlooked in emergencies. Its pathophysiology differs from that of allergic reactions such as anaphylaxis, making differentiation essential. We aimed to report two cases of KS that progressed to cardiac arrest, following the use of contrast medium. In both cases, extracorporeal membrane oxygenation (ECMO) was used for resuscitation, and coronary artery bypass graft surgery was planned for coronary artery reconstruction. ECMO should be considered in severe cases of KS, and clinicians should recognize that contrast‐induced KS can be life‐threatening.

## Key Clinical Message

Kounis syndrome, triggered by contrast medium, can lead to life‐threatening cardiac events. Early extracorporeal membrane oxygenation is crucial for resuscitation in severe cases, highlighting the need for awareness and prompt intervention in such emergencies.

## 1 Introduction

Kounis syndrome (KS), also known as allergic angina or myocardial infarction, is an immune‐mediated acute coronary artery disease [1]. KS is classified into three types: type I, caused by coronary spasms; type II, associated with plaque disruption; and type III, associated with coronary stents [2, 3]. The reported incidence in patients hospitalized for allergic symptoms is 2%–3.4% [4, 5, 6]; however, this rate may be underestimated. The in‐hospital mortality rate for KS is approximately 10 times higher than that for admitted patients with allergic symptoms [7]. Contrast‐induced KS is often severe and carries a higher mortality rate [8]. When patients with KS develop circulatory collapse, prompt initiation of extracorporeal membrane oxygenation (ECMO) may be required to prevent mortality. We aimed to report two cases of contrast‐induced KS in which the patients developed defibrillation‐resistant ventricular fibrillation (VF) and required ECMO support for survival.

## 2 Case History/Examination

In accordance with our institution's policies and standard publishing guidelines, approval by an institutional ethics committee was not required for this single case report describing routine clinical practice. The patient provided written informed consent for publication of all case details and images. The timeline for both cases is shown in Figure S1.

### 2.1 Case 1

A 58‐year‐old man with a history of arteriosclerosis obliterans, no known allergies, and no coronary artery disease was undergoing dialysis for diabetic nephropathy. He presented to the emergency department with an obstructed shunt and was admitted for vascular access intervention (shunt dilation therapy). A sheath was inserted into the radial artery, and 7 mL of iopamidol was administered.

**FIGURE 1:**
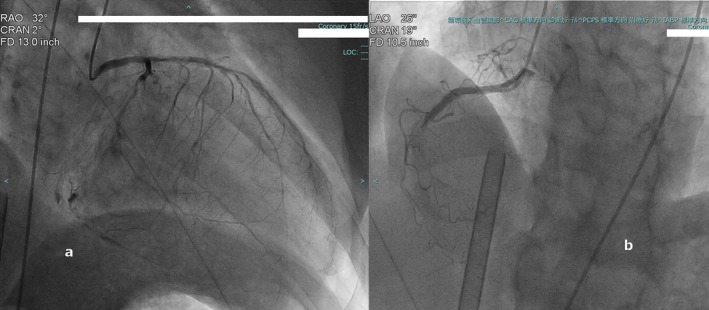
Left heart catheterization in Patient 1 shows severe stenosis of the proximal left anterior descending artery, diffuse stenosis of the left circumflex artery (a), and in‐stent occlusion of the right coronary artery (b).

Shortly afterward, following multiple sneezes, his blood pressure suddenly dropped. The patient was unresponsive to 20 mg of famotidine, 5 mg of chlorpheniramine, or intravenous fluid loading. He lost consciousness and was administered 0.3 mg adrenaline intramuscularly. However, his blood pressure dropped further, from 106/61 to 54/28 mmHg. Advanced Cardiovascular Life Support (ACLS) protocols were initiated; however, the patient developed VF, resistant to multiple defibrillation attempts at 150 J. Therefore, the resuscitation strategy was escalated to extracorporeal life support (ECLS); the patient was intubated, and ECMO was initiated via the femoral vessels. After administering 300 mg of amiodarone and performing additional defibrillation, a sinus rhythm (SR) was restored. When the contrast medium was changed to iodixanol, coronary angiography revealed complete occlusion of the right coronary artery (RCA), severe stenosis of the proximal left anterior descending (LAD) artery, and diffuse severe stenosis of the left circumflex artery (Figure 1).

**FIGURE 2:**
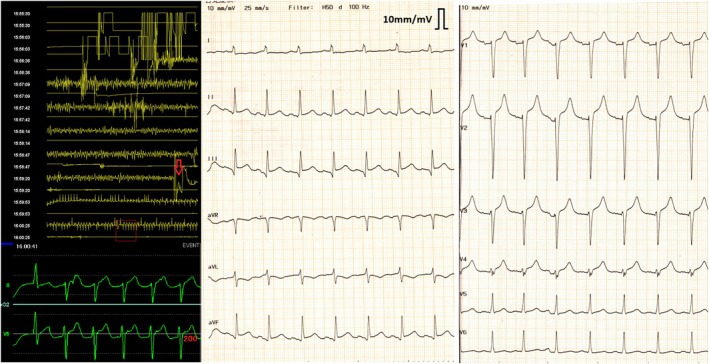
Left: At the point indicated by the red arrow, defibrillation restored sinus rhythm, ST elevation was observed in leads II and V5. Right: Electrocardiography at ICU admission reveals ST elevation in leads II, III, aVF, and V2–V4, indicating ischemia in both anterior and inferior locations. ICU, intensive care unit.

An intra‐aortic balloon pump (IABP) was inserted, and the patient was transferred to the intensive care unit (ICU). Figure 2 displays an electrocardiogram (ECG).

Transthoracic echocardiography demonstrated reduced contractility, a left ventricular ejection fraction (EF) of 35%, and decreased wall motion, primarily in the left ventricular anterior wall to the ventricular septum. Laboratory results at the time of cardiac arrest showed severe acidemia (pH: 6.95; base excess: −22 mmol/L). The white blood cell count was 4.87 × 10^3^/μL (reference range: 3.5–6.5 × 10^3^/μL), with neutrophils at 56.7% (reference range: 40.0%–70.0%) and eosinophils at 0.2% (reference range: 2.0%–9.0%). The maximum serum creatine kinase‐MB (CK‐MB) and troponin T levels were 0.26 and 76 ng/mL (reference ranges: < 5 and 0.01 ng/mL), respectively, on the first day of ICU admission.

In the ICU, a dialysis catheter was inserted through the right internal jugular vein, and continuous hemodialysis and filtration (CHDF) was initiated to correct the acid–base equilibrium and electrolyte imbalance. The ECMO and IABP were weaned on days 3 and 4, respectively. The patient transitioned from CHDF to hemodialysis on day 7 with improved cardiovascular stability. Ventilator weaning was delayed due to ventilator‐associated pneumonia but was completed on day 13. Coronary artery bypass graft surgery (CABG) was performed successfully on day 19, and the patient was transferred from the ICU to the ward on day 24. The patient was discharged from the hospital 31 days later without any apparent neurological complications and returned home.

### 2.2 Case 2

**FIGURE 3:**
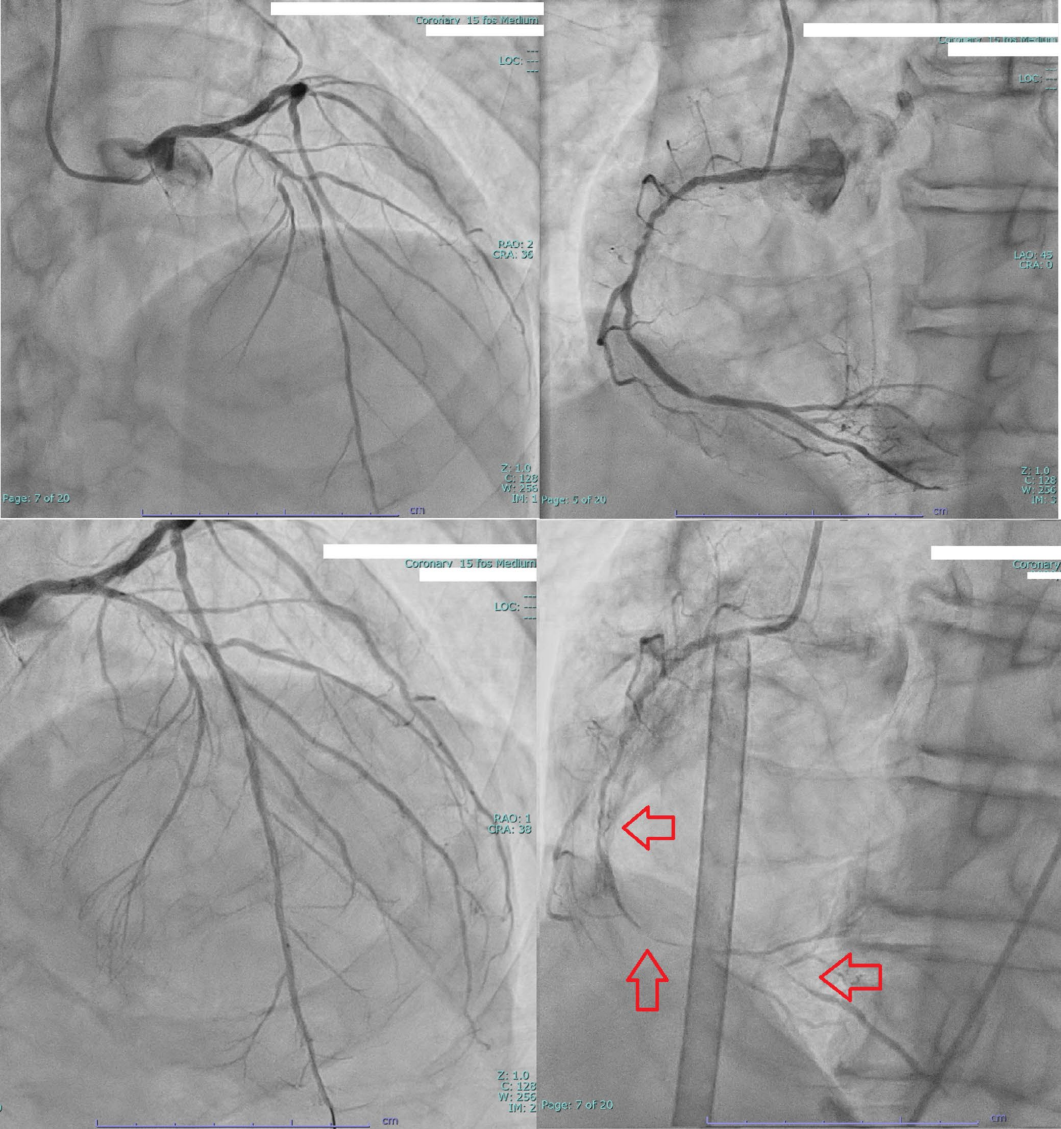
Left heart catheter images for Patient 2. The upper two images show views before the procedure, and the lower two images show views after contrast agent injection. Coronary artery spasm (Red arrow) is observed, particularly prominent in the right coronary artery.

A 79‐year‐old man with grade 5 chronic kidney disease and a history of multiple percutaneous coronary interventions (PCI) in the LAD and left circumflex arteries was admitted for creation of an upper‐extremity shunt to facilitate maintenance dialysis. However, 6 days after admission, he underwent PCI on a standby basis because of preoperative findings of poor cardiac function (left ventricular EF 27.1%) and significant stenosis in the RCA and LAD. Following iopamidol injection, the patient developed itching in the back, hypotension (from 117/71 to 49/23 mmHg), and ST‐segment elevation on ECG. He received 0.5 mg intramuscular adrenaline; however, his blood pressure did not normalize, leading to the initiation of ACLS protocols. The patient was intubated and progressed from pulseless electrical activity to VF, which was resistant to multiple defibrillation attempts at 150 J. Therefore, the resuscitation strategy was escalated to ECLS, and ECMO was initiated through the femoral vessels. Hydrocortisone (300 mg) was administered, and coronary angiography revealed coronary spasms mainly in the RCA (Figure 3).

**FIGURE 4:**
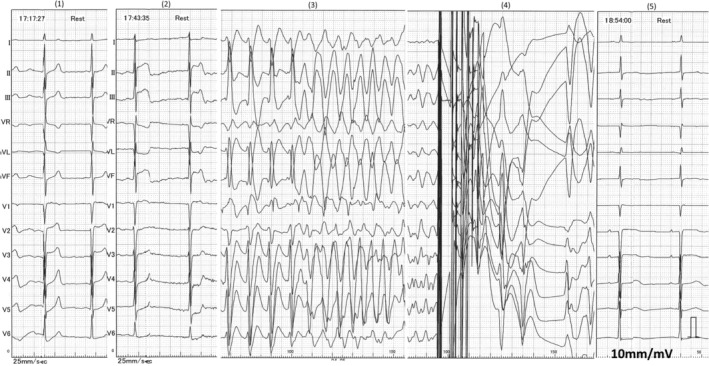
The electrocardiogram of Patient 2 is shown in chronological order from (1) to (5). (2) shows ST elevation in leads II, III, and aVF, followed by (3) showing ventricular fibrillation (VF). (4) shows restoration of sinus rhythm following defibrillation, and (5) shows no obvious ischemic findings.

Direct injection of isosorbide nitrate into the coronary arteries relieved the spasms. After 300 mg of amiodarone and defibrillation at 150 J, SR was restored. Upon returning to SR, ST elevation disappeared (Figure 3). An IABP was inserted, and the patient was transferred to the ICU after insertion of a dialysis catheter. On ICU day 2, the maximum serum CK‐MB and troponin T levels were 3.8 and 0.16 ng/mL, respectively. The white blood cell count was 6.60 × 10^3^/μL (reference range: 3.5–6.5 × 10^3^/μL), with 84.6% neutrophils (reference range: 40.0%–70.0%) and 1.5% eosinophils (reference range: 2.0%–9.0%). ECGs during VF and after return to SR are shown in Figure 4.

We monitored the patient's cardiac function after admission to the ICU. CHDF was performed to correct acid–base equilibrium, electrolyte disturbances, and fluid overload caused by resuscitation. The patient was weaned from ECMO and IABP on day 5 and transitioned from CHDF to hemodialysis on day 6, with improvement in cardiovascular status. He was extubated on day 8. CABG was planned to address pre‐existing coronary artery lesions that predated this episode, and the patient was transferred from the ICU to the ward on day 14. However, he developed a lymphatic fistula and an infection at the ECMO insertion site while in the ward. Despite treatment with antibiotics and debridement, the infection persisted, and the patient died 39 days after transfer from the ICU.

## 3 Diagnostics and Discussion

KS was first reported in 1991 by Kounis and Zavras as an acute coronary syndrome (ACS) caused by an allergic reaction. It is also referred to as *allergic myocardial infarction* or *allergic angina* [9]. Various factors, including drugs, environmental exposure, and certain medical conditions, can trigger KS [10]. Contrast medium accounts for a relatively small portion of causative agents [1]; however, it is associated with greater severity and higher mortality [8].

At present, three distinct subtypes of Kounis syndrome have been reported. Type I accounts for 72.6% of all cases, where the release of inflammatory mediators induces coronary artery spasm. In type II (22.3%), the release of inflammatory mediators induces plaque erosion or rupture. Type III accounts for only 5.1% of all cases and is thought to cause coronary stent thrombosis due to an allergic reaction, with histological evidence of mast cells and eosinophils in aspirated thrombi [1]. Case 1 corresponds to type II or III, and Case 2 likely corresponds to type I. This is because coronary angiography findings revealed atherosclerotic lesions and coronary vasospasm, respectively.

In the cases presented herein, KS was attributed to the contrast medium. Several studies have described PCI after steroid administration as a form of coronary artery reconstruction in KS [8]. However, to the best of our knowledge, this is the first report of CABG following KS. Both Cases 1 and 2 presented with lesions requiring revascularization; however, both patients experienced episodes of complete circulatory collapse induced by contrast agents, necessitating avoidance of further contrast use. Therefore, CABG, which avoids the need for contrast agents, was selected. In Case 1, the contrast agent was changed from iopamidol to iodixanol to avoid an allergic reaction and reduce the irritant reaction. Iopamidol is a low‐osmolality contrast medium (LOCM), while iodixanol is an isoosmolar contrast medium (IOCM). IOCMs have been suggested to have a lower potential than LOCMs to induce KS [8].

KS is difficult to manage because no standard diagnostic criteria exist, and treatment must address both allergic reactions and ACS. Anti‐allergy drugs can worsen ACS; for example, epinephrine may worsen coronary artery spasms through its alpha‐ and beta‐adrenergic effects and may exacerbate tachycardia and hypotension. Alternatives include glucagon or antihistamines. In contrast, drugs administered to treat ACS can worsen allergic symptoms. Beta‐blockers may intensify coronary spasms, and nitroglycerin may further worsen hypotension and tachycardia. Opioids such as morphine, codeine, and meperidine, used for chest pain, can induce mast cell degranulation and exacerbate allergic reactions; therefore, they should be administered with caution in patients with KS. Conversely, fentanyl and its derivatives are weak mast cell inducers. Early initiation of ECMO during resuscitation may help overcome these therapeutic conflicts.

In our patients, ECMO was initiated immediately. Because their circulation had collapsed and they presented with defibrillation‐resistant VF, ECMO was the only available means of life support. Both patients survived and were discharged from the ICU. As both were undergoing maintenance dialysis, their low circulating plasma volume likely contributed to circulatory collapse. To date, only three cases of ECMO treatment for KS have been reported [11, 12, 13]. ECMO is considered more effective than manual chest compressions for maintaining coronary circulation and facilitating cardiac recovery [14]. In addition, a patient's condition may improve through rapid removal of contrast medium with blood purification therapy in the ICU [15]. In Case 2, however, we were unable to proceed to CABG due to failure to control the infection. ECMO is useful but attention must be paid to bleeding and infection control at the puncture site, especially in such vulnerable patients.

This case series has some limitations. First, allergy‐related biomarkers such as IgE and tryptase could not be measured in both cases; besides, in Case 2, coronary angiography images obtained after isosorbide nitrate administration were not preserved, making it difficult to compare the images before and after drug administration. Second, only two cases were presented in this case series. Further accumulation of cases is needed to establish optimal diagnostic and treatment strategies.

## 4 Conclusion

We reported two cases of KS induced by contrast medium. Establishing diagnostic criteria for KS is urgently needed. Because contrast‐induced KS tends to be severe, extracorporeal cardiopulmonary resuscitation should be considered as a potentially effective intervention based on the patient's condition.

## Author Contributions

**Kiichi Yano:** investigation, writing – original draft. **Yu Asami:** conceptualization, writing – review and editing. **Takumi Inoue:** investigation, writing – original draft. **Emiko Takego:** conceptualization, writing – review and editing. **Kenji Tamai:** writing – review and editing. **Hiroyuki Takahashi:** supervision, writing – review and editing.

## Funding

The authors have nothing to report.

## Consent

Written informed consent was obtained from the patients for the publication of this case series and any accompanying images.

## Conflicts of Interest

The authors declare no conflicts of interest.

## Supporting information


**Figure S1:** Clinical courses of the two patients over time.

## Data Availability

The data that support the findings of this study are available from the corresponding author upon reasonable request.
