# Supplementary figures and images for: Two Cases of Kounis Syndrome Caused by Contrast Medium: Early Extracorporeal Membrane Oxygenation
Source: Clin Case Rep. 2026 Apr 28;14(5):e72635. doi: 10.1002/ccr3.72635 (PMC13124663; doi:10.1002/ccr3.72635)

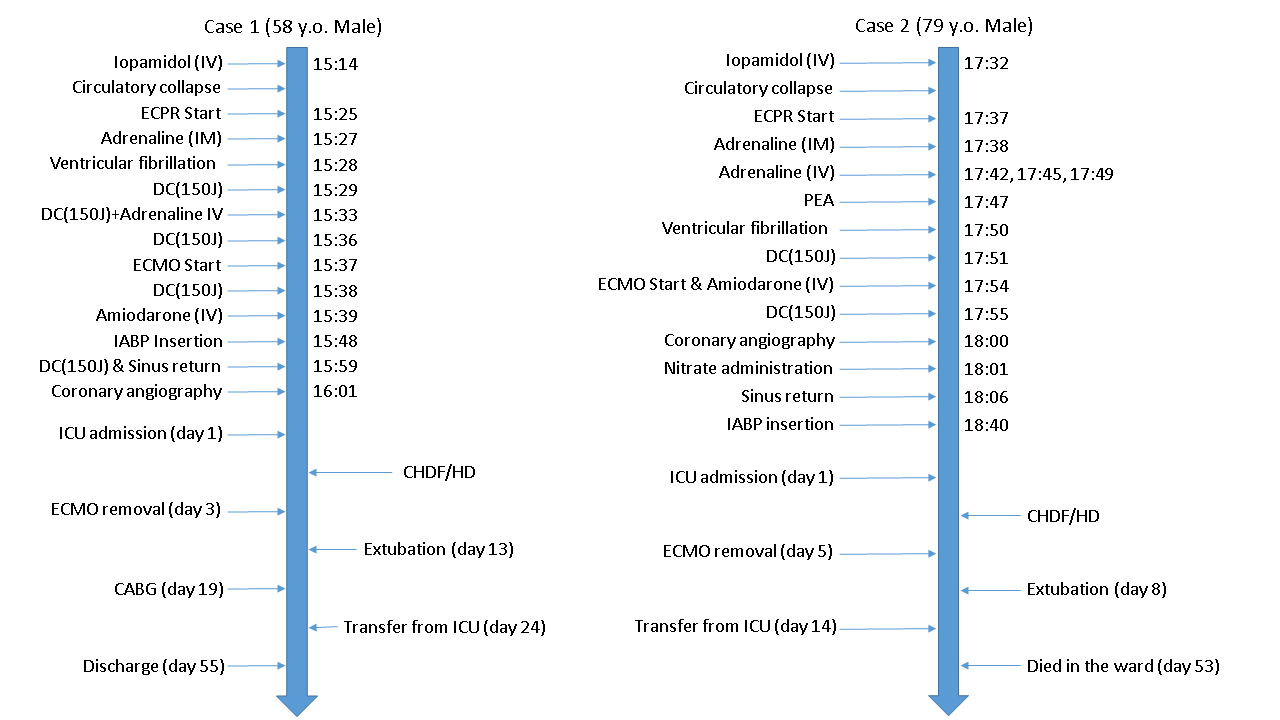

Supplement: Supplementary file 1 — Figure S1: Clinical courses of the two patients over time. [file CCR3-14-e72635-s001.tif]
